# Clinical Metabolomics: The New Metabolic Window for Inborn Errors of Metabolism Investigations in the Post-Genomic Era

**DOI:** 10.3390/ijms17071167

**Published:** 2016-07-20

**Authors:** Abdellah Tebani, Lenaig Abily-Donval, Carlos Afonso, Stéphane Marret, Soumeya Bekri

**Affiliations:** 1 Department of Metabolic Biochemistry, Rouen University Hospital, Rouen 76031, France; abdellah.tebani@chu-rouen.fr; 2 Normandie Univ, UNIROUEN, INSERM, CHU Rouen, IRIB, Laboratoire NeoVasc ERI28, Rouen 76000, France; lenaig.abily-donval@chu-rouen.fr (L.A.-D.); stephane.marret@chu-rouen.fr (S.M.); 3 Normandie Univ, UNIROUEN, INSA Rouen, CNRS, COBRA, Rouen 76000, France; carlos.afonso@univ-rouen.fr; 4 Department of Neonatal Pediatrics and Intensive Care, Rouen University Hospital, Rouen 76031, France

**Keywords:** metabolomics, inborn errors of metabolism, screening, diagnosis, systems medicine, precision medicine

## Abstract

Inborn errors of metabolism (IEM) represent a group of about 500 rare genetic diseases with an overall estimated incidence of 1/2500. The diversity of metabolic pathways involved explains the difficulties in establishing their diagnosis. However, early diagnosis is usually mandatory for successful treatment. Given the considerable clinical overlap between some inborn errors, biochemical and molecular tests are crucial in making a diagnosis. Conventional biological diagnosis procedures are based on a time-consuming series of sequential and segmented biochemical tests. The rise of “omic” technologies offers holistic views of the basic molecules that build a biological system at different levels. Metabolomics is the most recent “omic” technology based on biochemical characterization of metabolites and their changes related to genetic and environmental factors. This review addresses the principles underlying metabolomics technologies that allow them to comprehensively assess an individual biochemical profile and their reported applications for IEM investigations in the precision medicine era.

## 1. Introduction

The new field of precision medicine is revolutionizing current medical practice and reshaping future medicine. Precision medicine aspires to put the patient as the central driver of healthcare by broadening biological knowledge and acknowledging the greate diversity of individuals [1]. It is well established that complex gene–environment interactions shape normal physiological and disease processes at both the individual and population scale. Predicting normal and pathological states in patients requires dynamic and systematic understanding of these interactions. Systems medicine is a new concept based on holistic approaches for disease diagnosis and monitoring. The basic idea of these approaches is that a complex system is more comprehensively understood if considered as a whole at both the spatial and temporal scales.

Inborn errors of metabolism (IEM) are an appropriate model for systems medicine studies because the biological basis underlying these diseases has been, at least partly, revealed. IEM represent a group of about 500 rare genetic diseases with an overall estimated incidence of 1/2500. Even though these disorders are individually rare, they are collectively more common and cause a significant childhood morbidity and mortality. IEM are genetic disorders resulting from defects in a given biochemical pathway due to the deficiency or abnormality of an enzyme, its cofactor, or a transporter, leading to an accumulation of a substrate or lack of the product. Hence, the diversity of metabolic pathways involved explains the difficulties in establishing a diagnosis.

Autosomal recessive transmission is most frequent, but autosomal dominant and X-linked disorders have also been described. IEM may involve mutations in mitochondrial DNA. The pathogenesis of IEM can be explained by mechanisms such as deficiency of an essential product or enzyme, systemic toxic effects of circulating metabolites, and activation or inhibition of alternative metabolism [2]. Based on these pathophysiological traits, several IEM therapies have been developed, including dietary restriction, toxic product clearance, or biotherapies (enzyme replacement and gene therapy) [3]. Initiating these treatments at birth or at early stages is usually mandatory for optimal patient management. The first description of these disorders was made by Sir Archibald Garrod [4], who initiated the “one gene–one disease” paradigm. However, there is a lack of genotype–phenotype correlation in IEM. Furthermore, for the same genetic variation, different phenotypes have been observed in the same family [2]. These observations challenge Garrod’s paradigm and suggest the influence of either genetic or environmental modifying factors. Thus, IEM are more than monogenic diseases, which adds another layer of complexity to disease characterization and diagnosis.

The rise of “omic” approaches, enabled by the tremendous technological shift in both multiscale biological information capture and data management, offers an amazing opportunity to provide new effective tools for screening, diagnosis, treatment, and monitoring of these diseases. Omic technologies offer global views on the basic molecules that build a biological system at the cell, tissue, or organism level. Primarily, they aim to recover, in an untargeted, unbiased, and hypothesis-free fashion, the biological information carried by genes (genomics), mRNA (transcriptomics), proteins (proteomics), and metabolites (metabolomics). These holistic strategies clearly contrast with conventional studies, which are mainly hypothesis-driven and reductionist. To truly understand disease processes, a global investigative approach needs to be applied at multiple biological informational levels.

Since the early days of medicine, the human body is viewed as a collection of separate and independent components, and thus, physicians typically treated disease by trying to identify the single abnormality related to a single component. This approach lacks contextual information which is vital for mechanistic understanding of pathophysiology and, thus, for designing treatment strategies [5,6]. Indeed, the complete characterization of a biological system should include a structural, an organizational pattern and a functional description [7]. The structure comprises the fundamental actor components (genes, proteins and metabolites). The organization pattern denotes how these actors are linked to each other and how they are organized topologically (e.g., linear or branched sequence of reactions) and morphologically (membrane-bound or functional compartmentalization). The function describes how the whole system behaves in space and time with regard to metabolic fluxes and response to stimuli [8,9,10].

Systems biology is a new scientific field that tries to achieve this systemic understanding of biology and to fill in the gap between information and context from a biological standpoint. Systems biology can be defined as a holistic and systemic analysis of complex system inter-connections and their functional interrelationships [11,12,13,14]. Two vital pillars supported the emergence of systems biology: data generation and data modeling. On the one hand, the surge of high-throughput omics technologies allowed the retrieval of a global and comprehensive biological information. On the other hand, the amazing development of computational capabilities allowed complicated systems modeling and convenient and intuitive visualization. Furthermore, these informatics advancements are crucial for comprehensive integration and insightful interpretation of the complex biological information [15,16,17].

The patient-centric approach is essential to achieve the promise of personal and stratified medicine. Indeed, unlike conventional medical biology practice based primarily on sequential studies of genes, proteins, and metabolites, the great challenge of modern biology is to apprehend a disease as a complex, integrated, and dynamic network. The dynamic view refers to the quantitative and qualitative assessment of changes and interactions between the different layers of the biological information [7,18,19,20,21]. The genetic classifications of disease are now well established, given the modern genomic tools that can provide rich information about large patient cohorts. However, other highly complementary approaches based on proteomic and metabolic information can help researchers to biochemically or physiologically contextualize the underlying genetic information, thus helping to get closer to the phenotype and allowing patient stratification [22]. Thanks to disruptive technological jumps, a revolutionary vision was pioneered by Lee Hood, who coined the term P4 medicine [19], which is aimed to be predictive, preventive, personalized, and participatory. This new shift defines a new healthcare strategy in which each person serves as his or her own control over time [23].

The omics surge presents an amazing opportunity to provide new innovative tools for rapid diagnosis of IEM. Furthermore, metabolomics approaches are relevant for IEM because their basic pathophysiology is tightly related to metabolism. These diseases present with nonspecific clinical symptoms and appropriate laboratory tests are crucial in making a diagnosis. However, conventional biological diagnosis procedures are based on a series of sequential and segmented biochemical tests on various separated analytical platforms. This approach is slow, time-consuming, and complex, whereas optimal patient management requires improved speed of biochemical tests to allow early diagnosis and better monitoring of IEM. To address this need of faster screening and diagnosis strategies, metabolic profiling is a promising candidate.

In this review, we describe basic principles underlying metabolic phenotyping and metabolomic approaches that can be used to comprehensively assess an individual biochemical profile and their reported applications in IEM. Data for this review were identified by searches of PubMed and references from relevant articles using the search terms “metabolomics”, “metabonomics”, “metabolic profiling”, “inborn errors of metabolism”, and “inherited metabolic diseases”.

## 2. Metabolomics

### 2.1. Metabolites and Metabolome

The idea behind metabolomics goes back to ancient Greece, where doctors used the organoleptic characteristics of urine to link them to different medical conditions. Urine sweetness has been used to detect high glucose in diabetes [24]. Such organoleptic features are, of course, metabolic in origin. The word metabolome was coined by Olivier et al. in 1998 and defined as the set of metabolites synthesized by an organism [25]. Metabolome refers to the comprehensive complement of all metabolites present in a given biological system, fluid, cell, or tissue [26]. Metabolites can be defined as organic small molecules involved in enzymatic reactions. Thus, metabolomics is one of the “omic” technologies based on biochemical and molecular characterizations of the metabolome and the changes in metabolites related to genetic, environmental, drug or dietary, and other factors.

Metabolomics allows researchers to characterize these interactions and to evaluate the biochemical mechanisms involved in such changes in a systematic fashion. Indeed, metabolites fulfill the key criterion in that they change rapidly in response to physiological changes and may generate vital information about biochemical pathways that are modified in patients and in treated patients. Hence, metabolic profiling is highly informative since metabolites act as substrates or products in biochemical metabolic pathways [22,27,28,29].

Metabolomics has found applications in many disease studies and in complex interacting systems [22]. The possibility of predicting drug effects from baseline metabolic profiles has been demonstrated and gave rise to pharmacometabonomics as a potential effector for patient stratification and personalized medicine [30,31,32,33,34,35]. It is possible that the future of IEM diagnosis may be found in the developing area of metabolomics by doing simultaneous quantitative metabolic profiling of many metabolites in biological fluids.

### 2.2. Analytical Strategies and Chemical Information Retrieval

#### 2.2.1. Biological Samples

For biological information recovery, metabolomics generally uses biofluids, cells, or tissue extracts as primary sources of metabolic fingerprint data. Compared with intact or extracted tissues, urine and serum or plasma are the most commonly studied biofluids in clinical practice, because they are easily obtained and prepared [36,37,38,39]. However, other specialized fluids could be used, including cerebrospinal fluid [40,41] or saliva [42,43,44] and even breath [45,46]. Dried blood (and other biofluids) spots samples (DBS) have also been investigated [47,48,49,50] and were shown to be an interesting alternative to conventional liquid samples for generating metabolite profiles. Given their practical advantages such as low volume, low cost, and handling convenience, DBS is gaining interest as a sampling support for metabolic profiling in IEM [47,51,52,53]. Of note, most metabolomics studies, particularly in clinical metabolomics, include data from a single biofluid, most often blood or urine. However, biochemical signature in a biofluid denotes complex interrelationships from different organs, which add another complexity layer for metabolomics data interpretation. This could be only understood by investigating pathophysiological states from a metabolic interactions perspective taking into account local metabolome specificities and their contribution to systemic metabolome. Different data-driven approaches have been described to handle these issues using multiple biofluids sampling and metabolomics data modeling [54,55].

#### 2.2.2. Analytical Technologies

The human metabolome is a complex, highly responsive, and dynamic system. Thus, it raises different analytical challenges compared to other omics analysis approaches that are based on profiling large molecules built with a simple and limited set of subunits, such as nucleotides for genomics and transcriptomics and amino acids for proteomics. Thus, for identification and functional analysis of DNA, RNAs and proteins, the order combination of the subunits is what matters. It is the order of subunits that embodies the observed complexity that carries the biological information. Sequencing technologies rely basically on an incremental detection of these subunits. Researchers must figure out the order of the subunits to decode the carried biological information [56]. However, the same sequencing approach cannot be used to analyze metabolites in complex biofluids, because the analytical challenge is not simply to crack the order code, as there is no obvious order.

To retrieve the metabolic information, the metabolome requires a more complex analysis of chemical mixtures that allows components to be individually and selectively differentiated, identified and measured across a wide qualitative and quantitative chemical space.

The diversity of the physicochemical properties of the various metabolites groups adds another layer of complexity to metabolomics studies. This supplemental challenge has been the key driver for the development of various analytical protocols and platforms. Indeed, scientists tackled this analytical challenge even before the term metabolomics was coined. The first scientific article about metabolomics was published by Pauling and colleagues, in which they described a method using gas chromatographic separation with flame ionization detection to analyze the breath [57]. The authors referred to orthomolecular medicine linking the detected biochemical signature to phenotypes.

Since then, huge development has been made. The mainly used metabolic profiling technologies are nuclear magnetic resonance (NMR) spectroscopy [58,59,60] and mass spectrometry (MS), either combined or not to a gas phase or liquid phase separation method [27,51]. These technologies are suitable for metabolomics studies because they deliver global, unbiased, and comprehensive chemical information from complex mixtures. For information recovery, the multivariate spectroscopic data produced are typically analyzed using chemometric techniques to identify informative metabolic combinations that can be used for either sample classification or global biomarker discovery [51,61,62]. NMR spectroscopy is rapid and nondestructive and has the advantage of being highly reproducible. It is a powerful spectroscopic technology that offers atom-centered information that is crucial for molecular structure elucidation [63]. High-resolution NMR using stronger magnetic fields or two-dimensional NMR allows higher information recovery. The major drawback of NMR is its lack of sensitivity. However, MS offers complementary molecular information and is, by far, more sensitive than NMR. Hence, it allows higher metabolome coverage. The use of separation methods coupled to MS, such as liquid chromatography [38,39], gas chromatography [64], or capillary electrophoresis [65], allows a molecular separation step before MS detection. This enhances sensitivity and the dynamic range and provides complementary molecular information using the separation dimension.

Recently, approaches using another gas phase separation, ion mobility spectrometry (IMS) [66], has been gaining interest in metabolomics [67,68,69,70,71,72,73]. Indeed, IMS is a well-established post-ionization separation method based on size, shape, and charge performed on a millisecond timescale, which represents an intermediate timescale between chromatography (seconds) and high-resolution MS detection (microseconds). Coupled with high-resolution mass spectrometry and chromatography (LC-IM-MS), IMS provides additional analyte selectivity without significantly compromising the speed of MS-based measurements. The MS dimension affords accurate mass information, while the IMS dimension provides molecular, structural, and conformational information through the determination of the ion collision cross section. Indeed, ion mobility spectrometry adds a separation dimension to the hybrid MS instruments allowing, thus, a more comprehensive analysis of complex biological mixtures [69,74,75,76,77]. Furthermore, accessing retention time, accurate mass, and collision cross section obtained by the combination of LC-IM-MS allows measurement integration, which enhances molecular identification and consequently biomarker discovery [78,79].

Fourier transform mass spectrometry is another group of ultra-high-resolution methods that offer the highest resolving power, resolution, and mass-to-charge ratio (*m*/*z*) measurement accuracy and, hence, better metabolome coverage [80]. However, given their high cost, these methods are limited to only a few research groups.

Recently, to increase the high-throughput of global metabolic profiling analysis, ambient ionization sources were introduced. They are capable of direct sampling for complex matrices under ambient conditions. For example, atmospheric solids analysis probe [81], desorption electrospray ionization (DESI) [82,83,84], and rapid evaporative ionization MS methods [85,86] have been demonstrated to provide real-time, interpretable MS data on biofluids and tissues, in vivo and ex vivo, and will certainly reshape the future for high-throughput real-time metabolome analysis. In many surgeries, it is often difficult to distinct visually between the healthy and diseased tissues, and this requires time-consuming biopsies and immuno-staining procedures to be performed by histopathologists during surgery. By eliminating this need for external tissue histotyping, the iKnife could open the way to true real-time precision surgery. For more details about the use of ambient MS in clinical diagnosis, refer to a recent and detailed review by Ifa et al. [87].

Table 1 presents a comparison between different analytical strategies used in metabolomics with potential interest for IEM. Given the already existing chemical biomarker infrastructure and growing adoption of MS in clinical laboratories, its relatively low cost compared to NMR instruments, and the analytical performance of current mass spectrometers in terms of sensitivity and resolution in particular, MS-based metabolomics is a very promising tool in clinical biochemistry in the near future [88].

### 2.3. Metabolomics Workflows: Targeted vs. Untargeted

Metabolomics analysis is typically described as two complementary analytical approaches: targeted and untargeted. The first one aims to define the metabolic profile of the groups to study; subsequently, multivariate statistical analysis is undertaken to define the discriminating metabolites (potential biomarkers) between groups. Second, predictive mathematical models based on multivariate statistical analysis can be built. These models predict a subsequent classification of unknown biological samples (e.g., healthy versus diseased, treated versus untreated). The targeted approach focuses on identifying and quantifying selected metabolites according to their involvement in a metabolic pathway or their specific chemical or biochemical proprieties.

**Figure 1:**
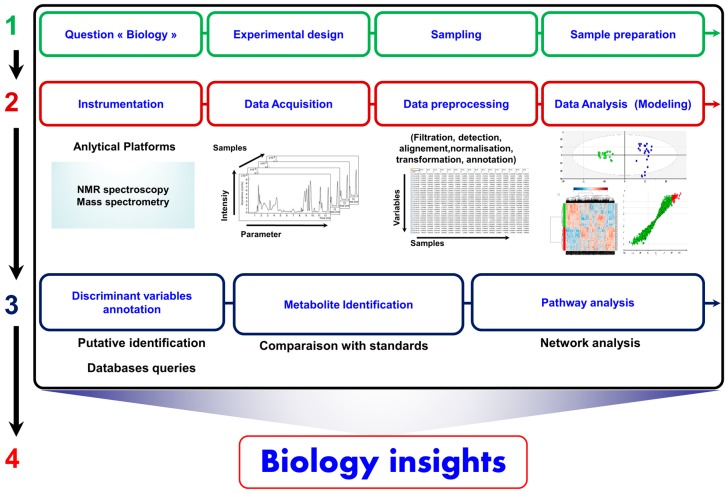
Translational metabolomics workflow.

In general, a metabolomic analysis involves mainly four steps. Step 1 is a preparatory step on both analytical and conceptual aspects. It is initiated by the biological question to consider and the definition of the study aim. It also defines the most informative biological matrix and the experimental design to implement. In addition, this step defines the appropriate sample preparation according to the considered analytical study. Step 2 includes analytical and instrumental strategy choices. During this step, data are collected and processed and then statistical analysis is performed. Step 3 involves the putative annotation, identification, and confirmation of the potential biomarkers generated by the data analysis. Chemical, biochemical, and spectral databases are queried. Step 4 aims to build a predictive mathematical model based on the identified biomarkers. This model is then validated analytically and clinically. This final step involves the integration of experimental data and their interpretation in the studied biological or clinical context [89]. Figure 1 illustrates the general workflow of translational metabolomics.

### 2.4. Data Analysis, Information Recovery, and the Curse of Dimensionality

Few highly reliable metabolites could be, at some extent, sufficient for diagnostic or monitoring purposes. However, a broader overview using more metabolites is more appropriate to assess, for example, a biochemical pathway. Thus, the choice of the most appropriate data modeling strategy is an important issue and is dependent on the underlying question to be addressed. In mechanistic studies, the structural data descriptions and the underlying extracted information yielded by the built model are more important than its predictive ability to classify new samples. However, in diagnosis applications, the predictive performances of the model are vital regarding samples classification. Hence, the clear and precise definition of the study aims has to be intelligible and purpose driven.

The analytical performance improvements associated with metabolomics platforms led to the generation of complex and high-dimensional datasets. Handling, in a smoothly high-throughput fashion, the huge amount of generated data is a very important issue for transforming the data into clinically actionable knowledge.

#### 2.4.1. Univariate Data Analysis

Metabolomics data analysis can be approached from a univariate perspective using traditional statistical methods that consider only one variable at a time. Univariate methods are common statistical analysis tools and their main advantage is the convenient use and interpretation. To assess the differences between two or more groups, parametric tests such as Student’s *t*-test and ANOVA are commonly applied, respectively. However, normality assumptions should be verified for consistent conclusions [90]. Otherwise, non-parametric test such as Mann–Whitney *U* test or Kruskal–Wallis one-way analysis of variance could be used if normality is not assumed. Another important issue is that applying multiple univariate tests in parallel to a high dimensional dataset raises the multiple testing problem. In metabolomics studies, a large number of features are simultaneously analyzed. Thus, the probability to find a statistically significant difference accidentally (i.e., true positive) is high. In order to handle this multiple testing issue, different correction methods could be used. Each method tries to balance between avoiding false metabolite associations (i.e., false positives) and discarding true associations (i.e., false negatives). In the Bonferroni correction, the significance level for a hypothesis is divided by the number of hypotheses tested simultaneously [90]. Hence, the Bonferroni correction is considered a stringent correction method. Other less conservative methods are available and are mostly based on the minimization of the false positives or false-discovery rate (FDR). FDR-based methods minimize the expected proportion of false positives on the total number of positives [91]. Gene expression microarray data analysis has matured most of these methods, where thousands of genes are simultaneously tested. Similarly, in untargeted metabolomics studies large sets of metabolites are measured in parallel. The use of less restrictive approaches such as FDR methods seems to be more useful.

Furthermore, it should be noted that potential confounding factors like gender, age or diet may affect the output results if not properly addressed. Furthermore, the main limit of these approaches is their lack of handling the correlations and interactions between the different metabolic features. Hence, advanced multivariate approaches are more suitable.

#### 2.4.2. Multivariate Data Analysis

Translating biological data into knowledge requires addressing biology as an informational science using tools that allow to track the information at large scales. To do so, an entire field was born “Bioinformatics” [92]. Bioinformatics can be defined as mean of conceptualizing biology in terms of molecules and by applying “informatics techniques” borrowed to disciplines such as applied mathematics, computer science and statistics to understand and organize the information related to these molecules, on a large scale. In short, bioinformatics is a management information system for a biological system [93].

The high-dimensionality of metabolic data requires adapted statistical tools to retrieve as much as possible chemical information from the data to translate it into biological knowledge. The major challenge is to reduce the dimensionality by selecting relevant signals from the noisy raw data. To achieve this goal, chemometric tools are widely used. Chemometrics is the science of extracting useful information from chemical systems using data-driven means [94]. It is inherently interdisciplinary, borrowing methods from data-analytic disciplines such as multivariate statistics, applied mathematics, and computer science. Thus, chemometrics is applied to solve both descriptive and predictive problems using biochemical data.

The data analysis methods are mainly divided into two types: unsupervised and supervised methods. The former are mainly exploratory, whereas the latter are explanatory and predictive. Unsupervised methods are used to analyze the behavior of the observations in the data set without taking into account any related outcome. Because there is no class labeling or response, the data set is considered as a collection of analogous objects. Unsupervised learning methods track patterns or clustering trends in the data to understand any spontaneous relationships between the samples. It can also highlight the variables that are responsible for these relationships. Based on effective visualization means, unsupervised learning helps to reveal categories of samples or variables that naturally cluster together based on their underlying similarities. In metabolomics data, it is the metabolic similarity that shapes the clustering. Principal component analysis [95] is a widely used pattern recognition method; it is a projection-based method that reduces the dimensionality of the data by creating components or latent variables. Principal component analysis allows a two- or three-dimensional visualization of the data. However, clustering methods aim to identify clusters in the dataset using similarity measures. A dendrogram or a heat map can be then formed to visualize the samples similarities. The commonly used clustering methods are k-means clustering [96], hierarchical cluster analysis [97], and self-organizing maps [98]. Correlation matrix could also be used to get an overview of the data. Because the main goal in metabolomics, especially in clinical context, is to differentiate between groups (healthy versus diseased, treated versus control), a sample can be classified according to its spectral patterns. The metabolic features responsible for the classification can then be identified. The metabolic features intensities in the dataset matrix can be considered as a multidimensional space of metabolites coordinates. Thus, each spectrum is a point in a multidimensional metabolic hyperspace.

In supervised methods, the multivariate datasets can be modeled so that the class label of separate samples known as a validation set can be predicted based on a series of mathematical models derived from the original data, namely the training set. Various supervised methods could be used in metabolomics, including partial least squares (PLS) methods such as PLS-Discriminant Analysis (PLS-DA) [99] and Orthogonal-PLS-DA (OPLS-DA) [100], as well as support vector machines [101]. Methods based on topology data analysis are gaining great interests and seem promising for data analysis because of their intrinsic flexibility and exploratory and predictive abilities [102]. It must be noted that the retrieved information from the raw data and the generated outputs are highly dependent on the chosen data analysis strategy. Hence, the aim of metabolomics research and the data analysis step are mutually dependent.

Of note, multivariate and univariate data analysis pipelines are not mutually exclusive and it is often recommended to use both to maximize the quality of the information extraction from metabolomics data.

For further details on data analysis techniques and tools in metabolomics, refer to recent reviews on this issue [103,104,105].

### 2.5. Pathway and Network Analysis: From Information to Knowledge

The integration of experimental data and computational tools is mandatory to understand complex biological systems. This gave birth to computational biology which could be divided into two distinct branches: knowledge discovery or data-mining, and simulation-based analysis. The former extracts the hidden patterns from huge amount of experimental data, generating hypotheses. However, the latter tests hypotheses with in silico experiments, providing predictions to be confirmed by in vitro and in vivo studies [9].

One of the biggest challenges of any metabolomics study is linking the identified metabolites to biology, which is a crucial step to move from biomarkers towards more mechanistic insights. To achieve this purpose, pathway and network analysis approaches aim to capitalize on the information generated by metabolomics studies to get insightful inference [106,107]. Both approaches exploit the interrelationships properties contained in the metabolomic data. Network modeling and pathway-mapping tools help to decipher metabolites interactions roles in a biological disturbance [107]. 

Metabolic pathways are sets of metabolites that are connected to the same biological process, and that are linked by one or multiple enzymatic reactions directly or indirectly. Biological databases are therefore seminal enablers providing rich information of different of metabolic pathways (Table 2). Indeed, pathway analysis (PA) uses prior biological knowledge to analyze metabolic patterns from an integrative point of view. Pathway-based methods are currently known as metabolite set enrichment analysis (MSEA), and are methodologically based on the gene set enrichment analysis (GSEA) approach, previously developed for pathway analysis of gene-expression data [108,109].

There are mainly three distinct methods to perform MSEA [108,116]:

Overrepresentation analysis (ORA): The basic hypothesis in this method is that relevant pathways can be detected if the proportion of differential expressed metabolites, within a given pathway, exceeds the proportion of metabolites that could be randomly expected. A hypergeometric test or a Fisher’s Exact test is used to evaluate the statistical significance of whether the metabolite belongs to the pathway. The final result from an ORA method consists in a list of the most relevant pathways, ranked by *p*-value and/or a multiple-hypothesis-test-corrected *p*-value. The ORA main advantage over non-knowledge-driven (i.e., purely data-driven) analysis is that it gives metabolomic data a biological context. This allows formulating a hypothesis that could subsequently be test experimentally. Hence, ORA turns data analysis into a knowledge generation cycle, proper of the Systems Biology approach. However, PA exhibits some limits. Due to the selected cut-off method for statistical significance potentially important components could be omitted in the analysis. Furthermore, PA assume that pathways are independent from each other, which is contrary to the admitted interaction and overlapping between pathways [108]. Other methods have been developed to overcome these limits.

Quantitative enrichment analysis (QEA): In this approach, the input data are a set of quantified metabolite from multiple samples. Thus, absolute concentrations are used. Enriched pathways can be identified using different approaches like the Wilcoxon-based test [117], globaltest [118] or globalAncova [119]. Enriched pathways include pathways where a set of metabolites that are significantly changed or pathways where a large number of metabolites that significantly changed [116,120].

Single-sample profiling (SSP): Unlike the previous methods that are designed for studies involving multiple samples, this method is used at the sample level. In this case, SSP requires a list of metabolite concentrations in biofluids (i.e., urine, blood and CSF), tissue, or cell type and a database with the normal concentration ranges of the chosen metabolites in the analyzed sample. Thus, SSP identifies, from the data, the set of metabolites presenting significantly different levels compared to the normal ranges [116,120].

For better interpretability of pathway analysis outputs, MSEA results could be combined with pathway topological analysis (PTA). PTA measures assess the impact of the disturbed metabolites within the pathway. First, single impacts are evaluated using the degree and betweenness network centrality measures of each metabolite. This represents the number of shortest paths passing through a certain node to estimate its centrality (importance). Subsequently, the overall impact (i.e., pathway impact) is calculated as the sum of the single impact measures of the disturbed metabolites normalized by the sum of the measures of the impact of all the metabolites within the considered pathway [121]. Indeed, changes in the most important nodes within a network generate a more significant impact on the system than changes in bordering or solitary nodes.

From a topological standpoint, a metabolic network can be considered as an interconnected ensemble of nodes presented by metabolites, and edges representing reactions catalyzed by enzymes. Thus, unlike PA, network analysis uses the high degree of correlation in metabolomics data to build metabolic networks that characterize the complex relationships the measured metabolites. Biological data exhibit a high level of correlation that exists between the different biological components (i.e., DNA, mRNAs, proteins and metabolites). Indeed, a given metabolite may be connected to different metabolic pathways and, thus, show correlation patterns. In other cases, the observed correlations may be due to other causes such as global changes (i.e., diurnal variation in time series studies) or specific changes due to the intrinsic variability of metabolomic data [54,122]. These patterns can provide valuable information about the underlying metabolic network associated to a specific biological process [54,123]. 

Based on the observed relationship patterns present in the experimental data, correlation-based methods allow building metabolic networks in which each metabolite represents a node. However, unlike the pathway analysis, the links between nodes denotes the level of mathematical correlation between each metabolites pair and called edge. High correlation coefficients are frequent in metabolomics data which is due to the presence of systemic associations [123]. Hence, using classical correlation coefficients leads to overcrowded networks. In addition, direct and indirect associations are not distinguished. To overcome this problem partial correlation could be used [54,123,124]. In partial correlation approach, the correlation between two metabolites is conditioned against the correlation with the remaining metabolites. Consequently, partial correlation allows discriminating between direct and indirect metabolite correlations. In this method, the link between two metabolites is scored according to the ratios differences between the corresponding metabolites in the two sample groups. Therefore, the related network topology is based on the metabolic differences between the two studied phenotypes. These data-driven strategies have been successfully applied for reconstruction of metabolic networks from metabolomics data [123,125,126].

Metabolite identification is a challenging and time consuming task. Thus, a novel approach, named Mummichog, has been proposed by Li et al. for network analysis. This method predicts biological activity directly from mass spectrometry based untargeted metabolomics data without a priori identification of metabolites. The idea behind this strategy is combining network analysis and metabolite prediction under the same computational framework reducing significantly the metabolomics workflow time. This method has been elegantly illustrated by exploring the activation of innate immune cells. It yielded that glutathione metabolism is modified by viral infection driven by constitutive nitric oxide synthases [127].

A wide variety of software tools are available to analyze metabolomic data at the pathway and network level. Table 3 presents different functional analysis tools for both pathway analysis and visualization.

Contextual interpretation is crucial to fully embrace the potential of metabolomics. Indeed, metabolites carry out precious contextual biological information. In a metabolic network, flux is defined as the rate (i.e., quantity per unit time) at which metabolites are converted or transported between different compartments [10]. Thus, metabolic fluxes, or fluxome, represent a unique and functional readout of the phenotype. The fluxome captures the metabolome in its ultimate functional interactions with the environment and the genome [10,142]. As such, the fluxome integrates information on different cellular processes, and hence it is a unique spatiotemporal phenotypic signature of cells. Thus, one or more metabolic fluxes could be altered in a metabolic disorder depending on the complexity of the disease [2]. Different strategies are used to translate metabolomics data into fluxomic insights by modeling of metabolic networks. The network modeling can be achieved using constraints of mass and charge conservation along with stoichiometric and thermodynamic ones [34,143,144,145]. Based on the stoichiometry of the reactants and products of biochemical reactions, flux balance analysis (FBA) can estimate metabolic fluxes without knowledge about the kinetics of the participating enzymes [10,142]. Recently, Cortassa et al. suggested a new approach, distinct from FBA or metabolic flux analysis, which takes into account kinetic mechanisms and regulatory interactions [146].

## 3. Potential Integration of Metabolomics in Laboratory Medicine Frameworks

Metabolites embody physiological end-points and regulatory processes directly connected to the fluxome. Hence, the metabolome is very time sensitive and is constantly changing. Therefore, changes in metabolite concentrations are usually more suitable to describe the biochemical state of a biological system. Because metabolomics is the ultimate expression of the genes’ influences and proteins’ use of metabolites, it offers a rich and tremendous view on the phenotype. Indeed, metabolites carry out precious contextual biological information that could be used to assess pathophysiological states.

Metabolic profiling as a diagnostic tool opens an informative metabolic window into disease, which makes metabolomics an appealing ally in disease diagnosis.

What makes metabolomics a key driver in the post-genomic era is its tight relationship with the phenotype, whether the phenotype is driven by a monogenic or a multifactorial complex condition. Linking metabolic profile modulation with particular genetic variation [126] and/or environmental factors such as the microbiome [147], diet [148], toxics [56], or therapies [34] offers an exciting opportunity to rationalize diagnostics and translate a more comprehensive information into clinical actionable knowledge. The above-cited factors that influence the metabolome, and then phenotype(s), remind us that assessing metabolites, as chemical supporters of life, is the core for knowledge building that will shape clinical decisions.

Early biochemists such as Cori, Warburg, Meyerhof, and Krebs made seminal contributions to map most fundamental aspects of metabolic pathways and physiology. Therefore, urine chemical properties guided early physicians in founding the concept of IEM [4]. Sir Garrod’s idea suggested that a biochemical fingerprint within biofluids was a product of human variation and, hence, could be a surrogate for distinct diseases. Garrod argued that the IEM that he was able to observe “were merely extreme examples of variations of chemical behavior which are probably everywhere present in minor degrees” [149]. In other words, he believed that there were phenotypes that could be associated with specific biochemicals. However, given the limited technical sensitivity back then, he was not able to affirm this idea. Recently, his hypothesis was elegantly confirmed with metabolomics approaches and metabolic modeling [126].

**Figure 2:**
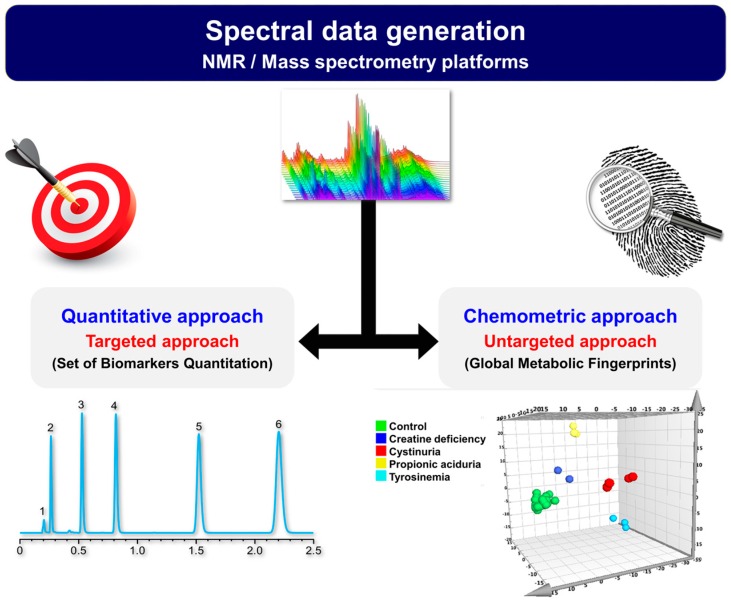
Clinical metabolomics implementation strategies.

With the expected improvements in the metabolic profiling scope and data quality, metabolomics is destined to play a major and disruptive role in the near future as an efficient screening and diagnostic tool [150]. There are mainly two ways that metabolomics could be implemented in clinical context and laboratory medicine: chemometrics or a quantitative approach. For the former, direct statistical analysis is applied to spectral patterns and signal intensity data, and identification of metabolites may be performed in the last step if needed. This method captures metabolic snapshots and builds pattern-recognition-based models using machine learning techniques to sort samples (subjects) according to their metabolic patterns. This approach is eloquently embodied by the intelligent scalpel (iKnife) introduced by Takatz et al. [85], which instantaneously classifies, in vivo and ex vivo, cancerous and noncancerous tissues. This compelling technology aims to help surgeons during cancer surgery [85,86]. In contrast, the quantitative approach targets a set of metabolites and then analyzes the quantitative data directly. This approach affords an absolute quantitation of a set of chosen metabolites (e.g., amino acids, carnitines and acylcarnitines, or organic acids). A multivariate predictive model can be built based on the absolute concentration of these metabolites to predict clinical status or intervention outcomes. Compared to quantitative metabolomics, the key advantage of chemometric profiling is its capability of automated and unbiased assessment of metabolomics data. However, it requires a large number of spectra and sample uniformity, which are less of a concern in quantitative metabolomics. Nevertheless, the multivariate data analysis strategies underlying the two strategies are quite similar. Figure 2 illustrates the two clinical workflows.

## 4. Applications of Metabolomics in Inborn Errors of Metabolism (IEM) Investigations

IEM being tightly connected with metabolism, the inherent pathophysiological changes are the main determinant of the metabolome and functional understanding of the disease. Hence, due to its intrinsic multidisciplinary nature, integrating biochemistry, analytical chemistry, advanced statistics and bioinformatics, metabolomics analysis represents a promising tool to achieve improved understanding and better diagnosis of IEM in the post-genomic and precision medicine era. For years, MS has been used in the assessment of inherited metabolic diseases. Several IEM are currently diagnosed using targeted MS-based metabolomics methods such as aminoacidopathies, organic acidurias, and fatty acid oxidation disorders [151,152,153,154,155]. Furthermore, MS is now widely implemented in IEM newborn screening national programs worldwide [156]. However, the combination of the already existing tools with data analysis strategies is compelling for better biological information recovery.

To assess different IEM including aminoacidopathies, organic aciduria, and mitochondrial disorders, Janeckova et al. used targeted analysis combined with multivariate data analysis. Their work showed how combining chemometrics modeling and absolute quantification are eloquently complementary in the assessment of IEMs [157]. Drecksen et al. used a similar approach to assess isovaleric aciduria (IVA) based on 86 urine samples: 10 untreated and 10 treated IVA cases, 12 heterozygotes, 22 children controls, and 32 adult controls. The work succeeded in producing a comprehensive profile of metabolites of practical significance in IVA [158]. Osterman et al. described a matrix-assisted laser desorption/ionization MS-based method for acylcarnitine and organic acid analysis on DBS. The method enabled the identification and quantification of metabolites involved in different organic aciduria and beta oxidation deficiencies [159]. Using targeted metabolomics addressing complex lipids, Fan and colleagues showed that some sphingolipids species were elevated in Niemann–Pick Type C1 subjects. These lipid biomarkers may be used for monitoring the efficacy of specific therapy [160]. 

Given the increasing potential of metabolomics in IEM, different groups published work regarding the usefulness of untargeted-metabolomics-based approaches in IEM in disease characterization, diagnosis, and biomarker discovery. For characterization of disease biosignatures, respiratory chain deficiencies have been investigated by several research groups to track specific metabolic signatures using metabolomics [161,162,163]. Wikoff et al. used MS-based untargeted metabolomics in plasma to characterize methylmalonic acidemia and propionic aciduria. Propionylcarnitine, a known biomarker, was retrieved using untargeted strategy which illustrates the potential of metabolic profiling in biomarker detection. Five additional plasma acylcarnitine metabolites presented significant differences between patients and control individuals. In addition, γ-butyrobetaine was highly increased in a subset of patients. This demonstrates that metabolomics can widen the range of metabolites associated with IEM [164]. Auray-Blais et al. used MS-based untargeted metabolomics for biomarker discovery in Fabry disease which led to the discovery of seven globotriaosylceramide (Gb3) analogues as biomarkers that are now suggested biomarkers for the screening and the follow-up of Fabry disease [61,62,165].

Sholmi et al. presented an elegant computational approach for assessing metabolic profiles of red blood cells enzyme deficiencies. The developed predictive method yielded biomarkers for red blood cells alterations and revealed a strong correlation with disrupted metabolic concentrations. Over 200 metabolites were identified as potential biomarkers due to 176 enzyme deficiencies. Furthermore, already known disease indicators were retrieved by the developed prediction method. Importantly, potential novel biomarkers were also predicted. This approach proved to dramatically increase biomarker discovery performance [166].

Because the metabolome is highly influenced by nutritional factors, diet monitoring also has been investigated using metabolomics. Phenylketonuria is an interesting example of diet monitoring in IEM. Using metabolomics, Mutze et al. showed that a long-term dietary fatty acid restriction influences mitochondrial beta-oxidation intermediates. No functional influence on unsaturated fatty acid metabolism and platelet aggregation in patients with phenylketonuria was detected [167].

Regarding the use of metabolomics platforms as a diagnosis tool, several teams proposed metabolomics workflows. Using NMR and DESI-MS methods, Pan et al. clearly discriminated six patients with IEMs from six controls based on their respective urine metabolic profiles, identifying argininosuccinic aciduria, classic homocystinuria, classic methylmalonic acidemia, maple syrup urine disease, phenylketonuria, and type II tyrosinemia [168]. Later, Denes et al. proposed a method based on high-resolution MS with high throughput using DBS and direct flow injection analysis. Their method has been tested on 500 controls and 66 abnormal samples and showed a clear discrimination of the various assessed metabolic diseases [51]. Ilya et al. also proposed another method based on high-resolution MS coupled to liquid chromatography for the assessment of IEM. Their method resolved highly polar as well as hydrophobic analytes under reverse-phase conditions, enabling analysis of a wide range of chemicals in an untargeted fashion. Their work provides a tailored high-resolution MS platform for IEM and covers various metabolites usually quantified by a combination of different separate instrumentation [169]. Miller et al. described a comprehensive global strategy to assess IEM using liquid chromatography and gas chromatography MS-based metabolomics platforms combining both targeted and untargeted analysis. In total, 120 plasma samples from patients with a confirmed IEM and those of 70 controls were assessed. This strategy allowed, elegantly, comprehensive pathway analysis that provides useful diagnostic information of IEM [170].

Regarding NMR-based platforms, Aygen and colleagues conducted a multi-center clinical study in 14 clinical centers in Turkey. Urine samples from 989 neonates were collected and investigated using NMR spectroscopy in two different laboratories to assess reproducibility. The objectives of their study were twofold: (1) to explore the metabolite variations to set pathological thresholds of specific metabolites in comparison with healthy neonates to develop predictive models; and (2) to build a NMR database from a healthy population of neonates for IEM metabolite identification [171].

## 5. Clinical and Translational Metabolomics Challenges

### 5.1. Metabolite Identification

Metabolite identification is the main bottleneck of metabolomics for large adoption in both translational and clinical context. Despite spectral information becomes available in the literature or in spectral databases, metabolites identification is still a challenging task [172]. To the best of our knowledge, there is no software currently available to fully and smoothly facilitate the identification process. Especially, the integration of NMR and MS data, which is essential for the reliable identification of metabolites. Furthermore, metabolite identification is mandatory for absolute quantitation especially in MS based methods requiring the use of labeled isotope. Thus, more efforts are needed to enhance this drawback of metabolomics.

### 5.2. Standardization and Harmonization

Standardization is a vital aspect for a wide spread of any new technology. Thus, for clinical metabolomics, harmonization of the sample preparation, processing, analysis and reporting using validated and standardized protocols is mandatory [173,174]. This is important since biological samples change over time. The lack of harmonization in protocols for sample handling, MS and NMR data generation and data reporting could lead to poor reproducibility and, thus, to data misinterpretation, particularly in population metabolic profiling. This is a fundamental obstacle for clinical translation. A definition of normal or reference samples is also important to build reference databases. This will rely on signatures derived from the complete characterization of the considered disease, IEM in the scope of this review. Finally, addressing these standardization issues is essential for regulatory compliance, which is a prerequisite for clinical implementation and adoption.

### 5.3. Automation, Data Visualization and Clinical Actionability

**Figure 3:**
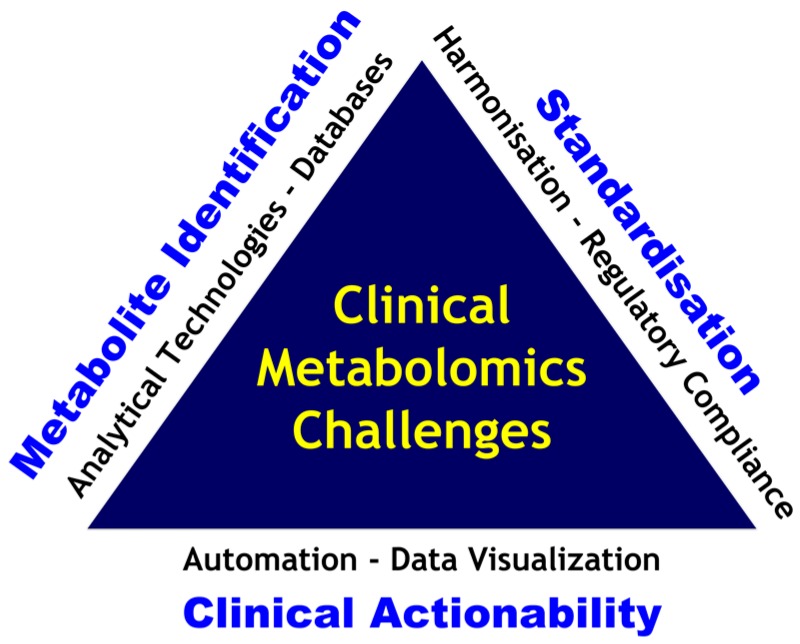
Metabolomics challenges for effective clinical implementation.

Automation at different stages, instrument-, pre- and post-analytic levels are a very important issue for large clinical adoption of any diagnostic innovation. Metabolomics workflow automation is a key enabler regarding high-throughput, reproducibility and reliability which are pillars of modern laboratory medicine practice. To address this limit, current efforts are promising like the iKnife, which would allow real-time cancer diagnosis [85] and breathomics strategies for lung and respiratory diseases based on breath signatures [72]. Data fusion and integration of omics and other biological and clinical data is another great challenge to fully unveil the potential of metabolomics [17,175]. With this regard, combining genomic and metabolic profiling information to enhance clinical diagnostics and to enable patient stratification and monitoring of interventional pathways patient journeys is a promising field [22,176]. The clinical actionability would involve advanced mathematical modeling of genomic and metabolic data sets in relation to patient clinical data using machine learning and expert systems. Intuitive visualization tools of the data in clinical accessible formats are needed to support effective clinical decision making. Figure 3 presents the main challenges in clinical metabolomics. 

## 6. Conclusions

It is common to perform early diagnosis of IEM by assessing specific metabolic biomarkers related to a genetic defect. However, the original paradigm of “one gene–one enzyme–one disease” is no longer viewed as a reality for IEM. The impact of an altered protein on metabolic flux is not easily predictable. Indeed, the metabolic pathways are not linear and metabolites are tightly linked with several interactions within a highly organized network [21,177]. Depending on the complexity of the disease, one primary metabolite flux or an entire network of metabolite fluxes might be affected [2,20]. Therefore, a complete contextual, multilayer, network-based functional overview is needed to effectively assess all the actors of a given pathway in a holistic fashion [8]. Systemic approaches are needed to understand IEM complexity and to effectively diagnose and treat them [21]. To achieve such a goal, metabolomics is a key driver in the systems medicine based strategy. The great potential of metabolomics integration with other omics data will allow systems biology and clinical data to be linked. This paves the way for a paradigm shift in medical practice from cohort evidence-based medicine to algorithm-based precision medicine. This will in turn enhance clinicians’ abilities to be more pre-emptive and thus, more efficient in handling IEM.

**Figure 4:**
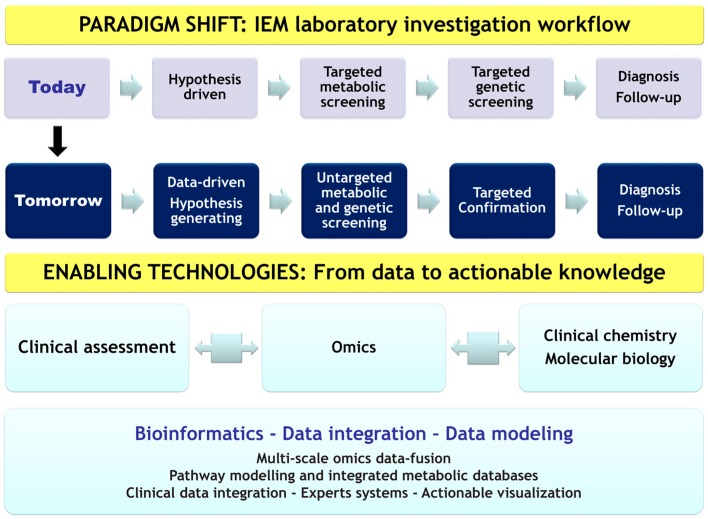
Paradigm shift in Inborn Errors of Metabolism diagnosis workflow. The change in molecular information recovery in laboratory investigation workflow is driven by advancing analytical technologies and bioinformatics systems for a more effective medical practice using an integrative computational framework. IEM: Inborn Errors of Metabolism.

Metabolomics is still in its infancy with regard to the investigation of IEM, and its great potential has yet to be explored worldwide at both the basic and clinical sides. Improving workflows for high-quality data acquisition, processing, and visualization is an important issue for effectively translating the biological information into actionable knowledge under clinically accessible formats for effective healthcare management. However, this innovative global approach also requires a paradigm shift in our practice at different levels. A complete change is needed in our screening and diagnosis strategies. Thus, a disruptive move from a hypothesis-driven approach to a more data-driven and hypothesis-generating approach is crucial to address the challenges of the post-genomic era. The core idea of the paradigm shift in IEM laboratory investigation is presented in Figure 4.

Furthermore, totally new investigative thinking is needed to transform all aspects of the laboratory medicine enterprise, including education, research, and healthcare. Upgrading medical practitioners’ skill sets on both the clinical and laboratory sides is needed to smoothly achieve the full potential of systems medicine. These skills integrate biology, computing and data analytics to develop common communication channels for more effective medical interactions. This ongoing high digitization of the individual biological and clinical information offers a tremendous and exciting opportunity to fully embrace the promising era of precision medicine.

## Figures and Tables

**Table 1:** Comparison of main metabolomics analytical technologies with particular potential in inborn errors of metabolism.

Platform	Technique	Identification Dimensions	Principle	Advantages	Limits
Nuclear Magnetic Resonance based methods (NMR)	1 Dimension 2 Dimensions	Chemical shift Chemical shift x Chemical shift	Uses interaction of spin active nuclei (^1^H, ^13^C, ^31^P) with electromagnetic fields which gives structural, chemical and molecular environment information	Nondestructive Highly reproducible Exact quantification possible Minimal sample preparation Molecular dynamic and compartmental information using diffusional methods Relative high throughput Availability of databases for identification	High instrumentation cost Overlap of metabolites Low sensitivity
Mass spectrometry based methods	Direct Injection (DI-MS)	*m*/*z*	Uses a nanospray source directly coupled to MS detector. It does not require chromatographic separation	Very high throughput High sensitivity No cross sample contamination No column carryover Low cost analysis Automated analysis Low sample volume requirement	Samples not recoverable (destructive) No retention time information which gives limited specificity Inability to separate isomers and isobaric species Subjected to significant ion suppression phenomenon High ionization discrimination (ESI)
	Liquid chromatography (LC-MS)	Time x *m*/*z*	Uses chromatographic columns that enables liquid phase chromatographic separation of molecules followed by MS detection (Suitable for polar to hydrophobic compounds)	Minimal sample preparation (protein precipitation or dilution of biological sample) High throughput capability UPLC can be coupled to any type of MS Flexibility in column chemistry widening the range of detectable compounds High sensitivity	Samples not recoverable (destructive) Very polar molecules need specific chromatographic conditions Retention times are highly dependent of exact chromatographic conditions Batch analysis Lack of large metabolite databases High ionization discrimination (ESI)
	Gas Chromatography (GC-MS)	Time x *m/z*	Uses chromatographic columns that enables gas phase chromatographic separation of molecules followed by MS detection (Suited for apolar and volatiles compounds)	Structure information obtained through in-source fragmentation Availability of universal databases for identification High sensitive Reproducible	Samples not recoverable (destructive) Requires higher sample preparation Only volatile compounds are detected Polar compounds need derivatization Low ionization discrimination
	Capillary Electrophoresis (CE-MS)	Time x *m*/*z*	Uses electrokinetic separation of polar molecules hyphenated with a mass spectrometry detector	Excellent for polar analysis in aqueous samples Measures inorganic and organic anions Low running costs	Samples not recoverable (destructive) Relatively low throughput profiling
	Ion Mobility (IM-MS)	Time x *m*/*z* (CCS x *m*/*z)*	Uses a uniform or periodic electric field and a buffer gas, to separate ions based on size and shape which is hyphenated with mass spectrometry	Very robust and reproducible (ability to determine Collision Cross Section which is a robust chemical descriptor) High peak capacity High selectivity Separation of isomeric and isobaric compounds Very high throughput	Samples not recoverable (destructive) CCS and mass are highly correlated parameters which limits the orthogonality of the method

**Table 2:** Biological databases.

Databases	Websites	Ref.
KEGG (Kyoto Encyclopedia of Genes and Genomes)	http://www.genome.jp/kegg	[110]
HumanCyc (Encylopedia of Human Metabolic Pathways)	http://humancyc.org	[111]
MetaCyc (Encyclopedia of Metabolic Pathways)	http://metacyc.org	[112]
Reactome (A Curated Knowledgebase of Pathways)	http://www.reactome.org	[113]
SMPDB (Small Molecule Pathway Database)	http://www.smpdb.ca	[114]
Virtual Metabolic Human Database	https://vmh.uni.lu	[106]
Wikipathways	http://www.wikipathways.org	[115]

**Table 3:** Functional analysis and biological interpretation tools.

Tools	Websites	Ref.
Pathway and Networks Analysis and Visualization		
BioCyc—Omics Viewer	http://biocyc.org	[128]
iPath	http://pathways.embl.de	[129]
Metscape	http://metscape.ncibi.org	[130]
Paintomics	http://www.paintomics.org	[131]
Pathos	http://motif.gla.ac.uk/Pathos	[132]
Pathvisio	http://www.pathvisio.org	[133]
VANTED	http://vanted.ipk-gatersleben.de	[134]
IMPaLA	http://impala.molgen.mpg.de	[135]
MBROLE 2.0	http://csbg.cnb.csic.es/mbrole2	[136]
MPEA	http://ekhidna.biocenter.helsinki.fi/poxo/mpea	[137]
Mummichog	http://clinicalmetabolomics.org/init/default/software	[127]
Multifunctional Tools		
MetaboAnlayst	http://www.metaboanalyst.com	[120]
XCMS online	https://xcmsonline.scripps.edu	[138]
MASSyPup	http://www.bioprocess.org/massypup	[139]
Workflow4Metabolomics	http://workflow4metabolomics.org	[140]
MetaboLyzer	https://sites.google.com/a/georgetown.edu/fornace-lab-informatics/home/metabolyzer	[141]

## References

[1] Collins F.S., Varmus H. (2015). A new initiative on precision medicine. N. Engl. J. Med..

[2] Lanpher B., Brunetti-Pierri N., Lee B. (2006). Inborn errors of metabolism: The flux from mendelian to complex diseases. Nat. Rev. Genet..

[3] Vernon H.J. (2015). Inborn errors of metabolism: Advances in diagnosis and therapy. JAMA Pediatr..

[4] Garrod A. (1902). The incidence of alkaptonuria: A study in chemical individuality. Lancet.

[5] Ahn A.C., Tewari M., Poon C.S., Phillips R.S. (2006). The limits of reductionism in medicine: Could systems biology offer an alternative?. PLoS Med..

[6] Regenmortel M.H.V.V. (2004). Reductionism and complexity in molecular biology. EMBO Rep..

[7] Aon M.A., Lloyd D., Saks V., Aon A.M., Saks V., Schlattner U. (2014). From physiology, genomes, systems, and self-organization to systems biology: The historical roots of a twenty-first century approach to complexity. Systems Biology of Metabolic and Signaling Networks: Energy, Mass and Information Transfer.

[8] Aon M.A. (2014). Complex systems biology of networks: The riddle and the challenge. Systems Biology of Metabolic and Signaling Networks.

[9] Kitano H. (2002). Computational systems biology. Nature.

[10] Aon M.A., Cortassa S. (2015). Systems biology of the fluxome. Processes.

[11] Weston A.D., Hood L. (2004). Systems biology, proteomics, and the future of health care: Toward predictive, preventative, and personalized medicine. J. Proteome Res..

[12] Ehrenberg M., Elf J., Aurell E., Sandberg R., Tegner J. (2003). Systems biology is taking off. Genome Res..

[13] Kitano H. (2002). Looking beyond the details: A rise in system-oriented approaches in genetics and molecular biology. Curr. Genet..

[14] Kitano H. (2002). Systems biology: A brief overview. Science.

[15] Tenenbaum J.D., Avillach P., Benham-Hutchins M., Breitenstein M.K., Crowgey E.L., Hoffman M.A., Jiang X., Madhavan S., Mattison J.E., Nagarajan R. (2016). An informatics research agenda to support precision medicine: Seven key areas. JAMIA.

[16] McMurry J., Kohler S., Balhoff J., Borromeo C., Brush M., Carbon S., Conlin T., Dunn N., Engelstad M., Foster E. (2016). Navigating the phenotype frontier: The monarch initiative. bioRxiv.

[17] Ritchie M.D., Holzinger E.R., Li R., Pendergrass S.A., Kim D. (2015). Methods of integrating data to uncover genotype-phenotype interactions. Nat. Rev. Genet..

[18] Sperisen P., Cominetti O., Martin F.-P.J. (2015). Longitudinal omics modeling and integration in clinical metabonomics research: Challenges in childhood metabolic health research. Front. Mol. Biosci..

[19] Hood L., Balling R., Auffray C. (2012). Revolutionizing medicine in the 21st century through systems approaches. Biotechnol. J..

[20] Cho D.-Y., Kim Y.-A., Przytycka T.M. (2012). Chapter 5: Network biology approach to complex diseases. PLoS Comput. Biol..

[21] Argmann C.A., Houten S.M., Zhu J., Schadt E.E. (2016). A next generation multiscale view of inborn errors of metabolism. Cell Metab..

[22] Nicholson J.K., Holmes E., Kinross J.M., Darzi A.W., Takats Z., Lindon J.C. (2012). Metabolic phenotyping in clinical and surgical environments. Nature.

[23] Chen R., Mias G.I., Li-Pook-Than J., Jiang L., Lam H.Y.K., Chen R., Miriami E., Karczewski K.J., Hariharan M., Dewey F.E. (2012). Personal omics profiling reveals dynamic molecular and medical phenotypes. Cell.

[24] Nicholson J.K., Lindon J.C. (2008). Systems biology: Metabonomics. Nature.

[25] Oliver S.G., Winson M.K., Kell D.B., Baganz F. (1998). Systematic functional analysis of the yeast genome. Trends Biotechnol..

[26] Nicholson J.K., Lindon J.C., Holmes E. (1999). “Metabonomics”: Understanding the metabolic responses of living systems to pathophysiological stimuli via multivariate statistical analysis of biological NMR spectroscopic data. Xenobiotica.

[27] Dunn W.B., Broadhurst D.I., Atherton H.J., Goodacre R., Griffin J.L. (2011). Systems level studies of mammalian metabolomes: The roles of mass spectrometry and nuclear magnetic resonance spectroscopy. Chem. Soc. Rev..

[28] Fiehn O. (2002). Metabolomics—The link between genotypes and phenotypes. Plant Mol. Biol..

[29] Holmes E., Wilson I.D., Nicholson J.K. (2008). Metabolic phenotyping in health and disease. Cell.

[30] Clayton T.A., Lindon J.C., Cloarec O., Antti H., Charuel C., Hanton G., Provost J.P., le Net J.L., Baker D., Walley R.J. (2006). Pharmaco-metabonomic phenotyping and personalized drug treatment. Nature.

[31] Kaddurah-Daouk R., Kristal B.S., Weinshilboum R.M. (2008). Metabolomics: A global biochemical approach to drug response and disease. Annu. Rev. Pharmacol. Toxicol..

[32] James L.P. (2013). Metabolomics: Integration of a new “omics” with clinical pharmacology. Clin. Pharmacol. Ther..

[33] Kaddurah-Daouk R., Weinshilboum R.M. (2014). Pharmacometabolomics: Implications for clinical pharmacology and systems pharmacology. Clin. Pharmacol. Ther..

[34] Kell D.B., Goodacre R. (2014). Metabolomics and systems pharmacology: Why and how to model the human metabolic network for drug discovery. Drug Discov. Today.

[35] Everett J.R. (2015). Pharmacometabonomics in humans: A new tool for personalized medicine. Pharmacogenomics.

[36] Dunn W.B., Broadhurst D., Begley P., Zelena E., Francis-McIntyre S., Anderson N., Brown M., Knowles J.D., Halsall A., Haselden J.N. (2011). Procedures for large-scale metabolic profiling of serum and plasma using gas chromatography and liquid chromatography coupled to mass spectrometry. Nat. Protoc..

[37] Nunes de Paiva M.J., Menezes H.C., de Lourdes Cardeal Z. (2014). Sampling and analysis of metabolomes in biological fluids. Analyst.

[38] Want E.J., Masson P., Michopoulos F., Wilson I.D., Theodoridis G., Plumb R.S., Shockcor J., Loftus N., Holmes E., Nicholson J.K. (2013). Global metabolic profiling of animal and human tissues via UPLC-MS. Nat. Protoc..

[39] Want E.J., Wilson I.D., Gika H., Theodoridis G., Plumb R.S., Shockcor J., Holmes E., Nicholson J.K. (2010). Global metabolic profiling procedures for urine using UPLC-MS. Nat. Protoc..

[40] Graham S.F., Chevallier O.P., Roberts D., Hölscher C., Elliott C.T., Green B.D. (2013). Investigation of the human brain metabolome to identify potential markers for early diagnosis and therapeutic targets of Alzheimer’s disease. Anal. Chem..

[41] Wuolikainen A., Hedenstrom M., Moritz T., Marklund S.L., Antti H., Andersen P.M. (2009). Optimization of procedures for collecting and storing of CSF for studying the metabolome in ALS. Amyotroph. Lateral Scler..

[42] Dame Z., Aziat F., Mandal R., Krishnamurthy R., Bouatra S., Borzouie S., Guo A., Sajed T., Deng L., Lin H. (2015). The human saliva metabolome. Metabolomics.

[43] Kawasaki G., Ichikawa Y., Yoshitomi I., Umeda M. (2015). Metabolomics of salivary biomarkers in yusho patients. Hukuoka Acta Med..

[44] Mikkonen J.J., Singh S.P., Herrala M., Lappalainen R., Myllymaa S., Kullaa A.M. (2015). Salivary metabolomics in the diagnosis of oral cancer and periodontal diseases. J. Periodontal Res..

[45] Bach J.-P., Gold M., Mengel D., Hattesohl A., Lubbe D., Schmid S., Tackenberg B., Rieke J., Maddula S., Baumbach J.I. (2015). Measuring compounds in exhaled air to detect Alzheimer’s disease and parkinson? S disease. PLoS ONE.

[46] Pijls K.E., Smolinska A., Jonkers D.M.A.E., Dallinga J.W., Masclee A.A.M., Koek G.H., van Schooten F.-J. (2016). A profile of volatile organic compounds in exhaled air as a potential non-invasive biomarker for liver cirrhosis. Sci. Rep..

[47] Koulman A., Prentice P., Wong M.C., Matthews L., Bond N.J., Eiden M., Griffin J.L., Dunger D.B. (2014). The development and validation of a fast and robust dried blood spot based lipid profiling method to study infant metabolism. Metabolomics.

[48] Wilson I. (2011). Global metabolic profiling (metabonomics/metabolomics) using dried blood spots: Advantages and pitfalls. Bioanalysis.

[49] Michopoulos F., Theodoridis G., Smith C.J., Wilson I.D. (2011). Metabolite profiles from dried blood spots for metabonomic studies using UPLC combined with orthogonal acceleration TOF-MS: Effects of different papers and sample storage stability. Bioanalysis.

[50] Prentice P., Turner C., Wong M.C.Y., Dalton R.N. (2013). Stability of metabolites in dried blood spots stored at different temperatures over a 2-year period. Bioanalysis.

[51] Denes J., Szabo E., Robinette S.L., Szatmari I., Szonyi L., Kreuder J.G., Rauterberg E.W., Takats Z. (2012). Metabonomics of newborn screening dried blood spot samples: A novel approach in the screening and diagnostics of inborn errors of metabolism. Anal. Chem..

[52] Wagner M., Tonoli D., Varesio E., Hopfgartner G. (2014). The use of mass spectrometry to analyze dried blood spots. Mass Spectrom. Rev..

[53] Oliveira R.V., Henion J., Wickremsinhe E.R. (2014). Automated high-capacity on-line extraction and bioanalysis of dried blood spot samples using liquid chromatography/high-resolution accurate mass spectrometry. Rapid Commun. Mass Spectrom..

[54] Do K.T., Kastenmüller G., Mook-Kanamori D.O., Yousri N.A., Theis F.J., Suhre K., Krumsiek J. (2015). Network-based approach for analyzing intra- and interfluid metabolite associations in human blood, urine, and saliva. J. Proteome Res..

[55] Torell F., Bennett K., Cereghini S., Rannar S., Lundstedt-Enkel K., Moritz T., Haumaitre C., Trygg J., Lundstedt T. (2015). Multi-organ contribution to the metabolic plasma profile using hierarchical modelling. PLoS ONE.

[56] Athersuch T. (2016). Metabolome analyses in exposome studies: Profiling methods for a vast chemical space. Arch. Biochem. Biophys..

[57] Pauling L., Robinson A.B., Teranishi R., Cary P. (1971). Quantitative analysis of urine vapor and breath by gas-liquid partition chromatography. Proc. Natl. Acad. Sci. USA.

[58] Jimenez B., Montoliu C., MacIntyre D.A., Serra M.A., Wassel A., Jover M., Romero-Gomez M., Rodrigo J.M., Pineda-Lucena A., Felipo V. (2010). Serum metabolic signature of minimal hepatic encephalopathy by (1) h-nuclear magnetic resonance. J. Proteome Res..

[59] Wijeyesekera A., Selman C., Barton R.H., Holmes E., Nicholson J.K., Withers D.J. (2012). Metabotyping of long-lived mice using 1 h NMR spectroscopy. J. Proteome Res..

[60] Larive C.K., Barding G.A., Dinges M.M. (2015). NMR spectroscopy for metabolomics and metabolic profiling. Anal. Chem..

[61] Auray-Blais C., Boutin M. (2012). Novel GB(3) isoforms detected in urine of fabry disease patients: A metabolomic study. Curr. Med. Chem..

[62] Manwaring V., Boutin M., Auray-Blais C. (2013). A metabolomic study to identify new globotriaosylceramide-related biomarkers in the plasma of fabry disease patients. Anal. Chem..

[63] Emwas A.-H., Salek R., Griffin J., Merzaban J. (2013). Nmr-based metabolomics in human disease diagnosis: Applications, limitations, and recommendations. Metabolomics.

[64] Chan E.C.Y., Pasikanti K.K., Nicholson J.K. (2011). Global urinary metabolic profiling procedures using gas chromatography-mass spectrometry. Nat. Protoc..

[65] Ramautar R., Somsen G.W., de Jong G.J. (2015). CE-MS for metabolomics: Developments and applications in the period 2012–2014. Electrophoresis.

[66] Hill H.H., Siems W.F., st Louis R.H., McMinn D.G. (1990). Ion mobility spectrometry. Anal. Chem..

[67] Paglia G., Angel P., Williams J.P., Richardson K., Olivos H.J., Thompson J.W., Menikarachchi L., Lai S., Walsh C., Moseley A. (2015). Ion mobility-derived collision cross section as an additional measure for lipid fingerprinting and identification. Anal. Chem..

[68] Maldini M., Natella F., Baima S., Morelli G., Scaccini C., Langridge J., Astarita G. (2015). Untargeted metabolomics reveals predominant alterations in lipid metabolism following light exposure in broccoli sprouts. Int. J. Mol. Sci..

[69] Paglia G., Williams J.P., Menikarachchi L., Thompson J.W., Tyldesley-Worster R., Halldórsson S., Rolfsson O., Moseley A., Grant D., Langridge J. (2014). Ion mobility derived collision cross sections to support metabolomics applications. Anal. Chem..

[70] Wickramasekara S.I., Zandkarimi F., Morre J., Kirkwood J., Legette L., Jiang Y., Gombart A.F., Stevens J.F., Maier C.S. (2013). Electrospray quadrupole travelling wave ion mobility time-of-flight mass spectrometry for the detection of plasma metabolome changes caused by xanthohumol in obese zucker (fa/fa) rats. Metabolites.

[71] Dwivedi P., Schultz A.J., Hill H.H. (2010). Metabolic profiling of human blood by high resolution ion mobility mass spectrometry (IM-MS). Int. J. Mass Spectrom..

[72] Hauschild A.C., Frisch T., Baumbach J.I., Baumbach J. (2015). Carotta: Revealing hidden confounder markers in metabolic breath profiles. Metabolites.

[73] Smolinska A., Hauschild A.C., Fijten R.R., Dallinga J.W., Baumbach J., van Schooten F.J. (2014). Current breathomics—A review on data pre-processing techniques and machine learning in metabolomics breath analysis. J. Breath Res..

[74] Fenn L., Kliman M., Mahsut A., Zhao S., McLean J. (2009). Characterizing ion mobility-mass spectrometry conformation space for the analysis of complex biological samples. Anal. Bioanal. Chem..

[75] Fenn L., McLean J. (2008). Biomolecular structural separations by ion mobility–mass spectrometry. Anal. Bioanal. Chem..

[76] Kliman M., May J.C., McLean J.A. (2011). Lipid analysis and lipidomics by structurally selective ion mobility-mass spectrometry. Biochim. Biophys. Acta.

[77] Tebani A., Schmitz-Afonso I., Rutledge D.N., Gonzalez B.J., Bekri S., Afonso C. (2016). Optimization of a liquid chromatography ion mobility-mass spectrometry method for untargeted metabolomics using experimental design and multivariate data analysis. Anal. Chim. Acta.

[78] May J.C., Goodwin C.R., McLean J.A. (2015). Ion mobility-mass spectrometry strategies for untargeted systems, synthetic, and chemical biology. Curr. Opin. Biotechnol..

[79] Sherrod S.D., McLean J.A. (2015). Systems-wide high-dimensional data acquisition and informatics using structural mass spectrometry strategies. Clin. Chem..

[80] Junot C., Madalinski G., Tabet J.C., Ezan E. (2010). Fourier transform mass spectrometry for metabolome analysis. Analyst.

[81] Twohig M., Shockcor J.P., Wilson I.D., Nicholson J.K., Plumb R.S. (2010). Use of an atmospheric solids analysis probe (ASAP) for high throughput screening of biological fluids: Preliminary applications on urine and bile. J. Proteome Res..

[82] Eberlin L.S., Norton I., Orringer D., Dunn I.F., Liu X., Ide J.L., Jarmusch A.K., Ligon K.L., Jolesz F.A., Golby A.J. (2013). Ambient mass spectrometry for the intraoperative molecular diagnosis of human brain tumors. Proc. Natl. Acad. Sci. USA.

[83] Ferreira C.R., Jarmusch A.K., Pirro V., Alfaro C.M., Gonzalez-Serrano A.F., Niemann H., Wheeler M.B., Rabel R.A., Hallett J.E., Houser R. (2015). Ambient ionisation mass spectrometry for lipid profiling and structural analysis of mammalian oocytes, preimplantation embryos and stem cells. Reprod. Fertil. Dev..

[84] Kerian K.S., Jarmusch A.K., Pirro V., Koch M.O., Masterson T.A., Cheng L., Cooks R.G. (2015). Differentiation of prostate cancer from normal tissue in radical prostatectomy specimens by desorption electrospray ionization and touch spray ionization mass spectrometry. Analyst.

[85] Balog J., Sasi-Szabo L., Kinross J., Lewis M.R., Muirhead L.J., Veselkov K., Mirnezami R., Dezso B., Damjanovich L., Darzi A. (2013). Intraoperative tissue identification using rapid evaporative ionization mass spectrometry. Sci. Transl. Med..

[86] Balog J., Kumar S., Alexander J., Golf O., Huang J., Wiggins T., Abbassi-Ghadi N., Enyedi A., Kacska S., Kinross J. (2015). In vivo endoscopic tissue identification by rapid evaporative ionization mass spectrometry (REIMS). Angew. Chem. Int. Ed. Engl..

[87] Ifa D.R., Eberlin L.S. (2016). Ambient ionization mass spectrometry for cancer diagnosis and surgical margin evaluation. Clin. Chem..

[88] Annesley T., Diamandis E., Bachmann L., Hanash S., Hart B., Javahery R., Singh R., Smith R. (2016). A spectrum of views on clinical mass spectrometry. Clin. Chem..

[89] Wishart D.S., Jewison T., Guo A.C., Wilson M., Knox C., Liu Y., Djoumbou Y., Mandal R., Aziat F., Dong E. (2013). Hmdb 3.0—The human metabolome database in 2013. Nucleic Acids Res..

[90] Broadhurst D.I., Kell D.B. (2006). Statistical strategies for avoiding false discoveries in metabolomics and related experiments. Metabolomics.

[91] Benjamini Y., Hochberg Y. (1995). Controlling the false discovery rate: A practical and powerful approach to multiple testing. J. R. Stat. Soc..

[92] Hogeweg P. (2011). The roots of bioinformatics in theoretical biology. PLoS Comput. Biol..

[93] Luscombe N.M., Greenbaum D., Gerstein M. (2001). What is bioinformatics? A proposed definition and overview of the field. Methods Inf. Med..

[94] Brereton R.G. (2014). A short history of chemometrics: A personal view. J. Chemom..

[95] Hotelling H. (1933). Analysis of a Complex of Statistical Variables into Principal Components.

[96] Hartigan J.A., Wong M.A. (1979). Algorithm as 136: A k-means clustering algorithm. J. R. Stat. Soc..

[97] Johnson S.C. (1967). Hierarchical clustering schemes. Psychometrika.

[98] Kohonen T. (1990). The self-organizing map. Proc. IEEE.

[99] Wold S., Sjöström M., Eriksson L. (2001). PLS-regression: A basic tool of chemometrics. Chemom. Intell. Lab. Syst..

[100] Trygg J., Wold S. (2002). Orthogonal projections to latent structures (O-PLS). J. Chemom..

[101] Cortes C., Vapnik V. (1995). Support-vector networks. Mach. Learn..

[102] Offroy M., Duponchel L. (2016). Topological data analysis: A promising big data exploration tool in biology, analytical chemistry and physical chemistry. Anal. Chim. Acta.

[103] Ren S., Hinzman A., Kang E., Szczesniak R., Lu L. (2015). Computational and statistical analysis of metabolomics data. Metabolomics.

[104] Gromski P.S., Muhamadali H., Ellis D.I., Xu Y., Correa E., Turner M.L., Goodacre R. (2015). A tutorial review: Metabolomics and partial least squares-discriminant analysis—A marriage of convenience or a shotgun wedding. Anal. Chim. Acta.

[105] Misra B.B., van der Hooft J.J. (2016). Updates in metabolomics tools and resources: 2014–2015. Electrophoresis.

[106] Thiele I., Swainston N., Fleming R.M., Hoppe A., Sahoo S., Aurich M.K., Haraldsdottir H., Mo M.L., Rolfsson O., Stobbe M.D. (2013). A community-driven global reconstruction of human metabolism. Nat. Biotechnol..

[107] Cazzaniga P., Damiani C., Besozzi D., Colombo R., Nobile M.S., Gaglio D., Pescini D., Molinari S., Mauri G., Alberghina L. (2014). Computational strategies for a system-level understanding of metabolism. Metabolites.

[108] Garcia-Campos M.A., Espinal-Enriquez J., Hernandez-Lemus E. (2015). Pathway analysis: State of the art. Front. Physiol..

[109] Khatri P., Sirota M., Butte A.J. (2012). Ten years of pathway analysis: Current approaches and outstanding challenges. PLoS Comput. Biol..

[110] Kanehisa M., Sato Y., Kawashima M., Furumichi M., Tanabe M. (2016). Kegg as a reference resource for gene and protein annotation. Nucleic Acids Res..

[111] Romero P., Wagg J., Green M.L., Kaiser D., Krummenacker M., Karp P.D. (2005). Computational prediction of human metabolic pathways from the complete human genome. Genome Biol..

[112] Caspi R., Foerster H., Fulcher C.A., Kaipa P., Krummenacker M., Latendresse M., Paley S., Rhee S.Y., Shearer A.G., Tissier C. (2008). The metacyc database of metabolic pathways and enzymes and the biocyc collection of pathway/genome databases. Nucleic Acids Res..

[113] Vastrik I., D’Eustachio P., Schmidt E., Gopinath G., Croft D., de Bono B., Gillespie M., Jassal B., Lewis S., Matthews L. (2007). Reactome: A knowledge base of biologic pathways and processes. Genome Biol..

[114] Jewison T., Su Y., Disfany F.M., Liang Y., Knox C., Maciejewski A., Poelzer J., Huynh J., Zhou Y., Arndt D. (2014). Smpdb 2.0: Big improvements to the small molecule pathway database. Nucleic Acids Res..

[115] Kelder T., van Iersel M.P., Hanspers K., Kutmon M., Conklin B.R., Evelo C.T., Pico A.R. (2012). Wikipathways: Building research communities on biological pathways. Nucleic Acids Res..

[116] Xia J., Wishart D.S. (2010). MSEA: A web-based tool to identify biologically meaningful patterns in quantitative metabolomic data. Nucleic Acids Res..

[117] Adjaye J., Huntriss J., Herwig R., BenKahla A., Brink T.C., Wierling C., Hultschig C., Groth D., Yaspo M.L., Picton H.M. (2005). Primary differentiation in the human blastocyst: Comparative molecular portraits of inner cell mass and trophectoderm cells. Stem Cells.

[118] Goeman J.J., van de Geer S.A., de Kort F., van Houwelingen H.C. (2004). A global test for groups of genes: Testing association with a clinical outcome. Bioinformatics.

[119] Hummel M., Meister R., Mansmann U. (2008). Globalancova: Exploration and assessment of gene group effects. Bioinformatics.

[120] Xia J., Sinelnikov I.V., Han B., Wishart D.S. (2015). Metaboanalyst 3.0—Making metabolomics more meaningful. Nucleic Acids Res..

[121] Xia J., Wishart D.S. (2010). METPA: A web-based metabolomics tool for pathway analysis and visualization. Bioinformatics.

[122] Steuer R. (2006). Review: On the analysis and interpretation of correlations in metabolomic data. Brief. Bioinform..

[123] Krumsiek J., Suhre K., Illig T., Adamski J., Theis F.J. (2011). Gaussian graphical modeling reconstructs pathway reactions from high-throughput metabolomics data. BMC Syst. Biol..

[124] Valcarcel B., Wurtz P., Seich al Basatena N.K., Tukiainen T., Kangas A.J., Soininen P., Jarvelin M.R., Ala-Korpela M., Ebbels T.M., de Iorio M. (2011). A differential network approach to exploring differences between biological states: An application to prediabetes. PLoS ONE.

[125] Bartel J., Krumsiek J., Schramm K., Adamski J., Gieger C., Herder C., Carstensen M., Peters A., Rathmann W., Roden M. (2015). The human blood metabolome-transcriptome interface. PLoS Genet..

[126] Shin S.Y., Fauman E.B., Petersen A.K., Krumsiek J., Santos R., Huang J., Arnold M., Erte I., Forgetta V., Yang T.P. (2014). An atlas of genetic influences on human blood metabolites. Nat. Genet..

[127] Li S., Park Y., Duraisingham S., Strobel F.H., Khan N., Soltow Q.A., Jones D.P., Pulendran B. (2013). Predicting network activity from high throughput metabolomics. PLoS Comput. Biol..

[128] Caspi R., Billington R., Ferrer L., Foerster H., Fulcher C.A., Keseler I.M., Kothari A., Krummenacker M., Latendresse M., Mueller L.A. (2016). The metacyc database of metabolic pathways and enzymes and the biocyc collection of pathway/genome databases. Nucleic Acids Res..

[129] Yamada T., Letunic I., Okuda S., Kanehisa M., Bork P. (2011). Ipath2.0: Interactive pathway explorer. Nucleic Acids Res..

[130] Karnovsky A., Weymouth T., Hull T., Tarcea V.G., Scardoni G., Laudanna C., Sartor M.A., Stringer K.A., Jagadish H.V., Burant C. (2012). Metscape 2 bioinformatics tool for the analysis and visualization of metabolomics and gene expression data. Bioinformatics.

[131] Garcia-Alcalde F., Garcia-Lopez F., Dopazo J., Conesa A. (2011). Paintomics: A web based tool for the joint visualization of transcriptomics and metabolomics data. Bioinformatics.

[132] Leader D.P., Burgess K., Creek D., Barrett M.P. (2011). Pathos: A web facility that uses metabolic maps to display experimental changes in metabolites identified by mass spectrometry. Rapid Commun. Mass Spectrom..

[133] Kutmon M., van Iersel M.P., Bohler A., Kelder T., Nunes N., Pico A.R., Evelo C.T. (2015). Pathvisio 3: An extendable pathway analysis toolbox. PLoS Comput. Biol..

[134] Rohn H., Junker A., Hartmann A., Grafahrend-Belau E., Treutler H., Klapperstück M., Czauderna T., Klukas C., Schreiber F. (2012). Vanted v2: A framework for systems biology applications. BMC Syst. Biol..

[135] Kamburov A., Cavill R., Ebbels T.M.D., Herwig R., Keun H.C. (2011). Integrated pathway-level analysis of transcriptomics and metabolomics data with impala. Bioinformatics.

[136] Lopez-Ibanez J., Pazos F., Chagoyen M. (2016). Mbrole 2.0-functional enrichment of chemical compounds. Nucleic Acids Res..

[137] Kankainen M., Gopalacharyulu P., Holm L., Oresic M. (2011). Mpea—Metabolite pathway enrichment analysis. Bioinformatics.

[138] Tautenhahn R., Patti G.J., Rinehart D., Siuzdak G. (2012). Xcms online: A web-based platform to process untargeted metabolomic data. Anal. Chem..

[139] Winkler R. (2015). An evolving computational platform for biological mass spectrometry: Workflows, statistics and data mining with massypup64. PeerJ.

[140] Giacomoni F., le Corguille G., Monsoor M., Landi M., Pericard P., Petera M., Duperier C., Tremblay-Franco M., Martin J.F., Jacob D. (2015). Workflow4metabolomics: A collaborative research infrastructure for computational metabolomics. Bioinformatics.

[141] Mak T.D., Laiakis E.C., Goudarzi M., Fornace A.J. (2014). Metabolyzer: A novel statistical workflow for analyzing postprocessed LC-MS metabolomics data. Anal. Chem..

[142] Cascante M., Marin S. (2008). Metabolomics and fluxomics approaches. Essays Biochem..

[143] Cortassa S., Aon M.A. (2012). Computational modeling of mitochondrial function. Methods Mol. Biol..

[144] Winter G., Kromer J.O. (2013). Fluxomics—Connecting “omics” analysis and phenotypes. Environ. Microbiol..

[145] Aurich M.K., Thiele I. (2016). Computational modeling of human metabolism and its application to systems biomedicine. Methods Mol. Biol..

[146] Cortassa S., Caceres V., Bell L.N., O’Rourke B., Paolocci N., Aon M.A. (2015). From metabolomics to fluxomics: A computational procedure to translate metabolite profiles into metabolic fluxes. Biophys. J..

[147] Cho I., Blaser M.J. (2012). The human microbiome: At the interface of health and disease. Nat. Rev. Genet..

[148] Holmes E., Loo R.L., Stamler J., Bictash M., Yap I.K., Chan Q., Ebbels T., de Iorio M., Brown I.J., Veselkov K.A. (2008). Human metabolic phenotype diversity and its association with diet and blood pressure. Nature.

[149] Garrod A.E. (1931). The Inborn Factors in Disease.

[150] Beebe K., Kennedy A.D. (2016). Sharpening precision medicine by a thorough interrogation of metabolic individuality. Comput. Struct. Biotechnol. J..

[151] Auray-Blais C., Maranda B., Lavoie P. (2014). High-throughput tandem mass spectrometry multiplex analysis for newborn urinary screening of creatine synthesis and transport disorders, triple H syndrome and otc deficiency. Clin. Chim. Acta.

[152] Pitt J.J. (2009). Principles and applications of liquid chromatography-mass spectrometry in clinical biochemistry. Clin. Biochem. Rev..

[153] Pitt J.J. (2010). Newborn screening. Clin. Biochem. Rev..

[154] Pitt J.J., Eggington M., Kahler S.G. (2002). Comprehensive screening of urine samples for inborn errors of metabolism by electrospray tandem mass spectrometry. Clin. Chem..

[155] Spacil Z., Tatipaka H., Barcenas M., Scott C.R., Turecek F., Gelb M.H. (2013). High-throughput assay of 9 lysosomal enzymes for newborn screening. Clin. Chem..

[156] Therrell B.L., Padilla C.D., Loeber J.G., Kneisser I., Saadallah A., Borrajo G.J., Adams J. (2015). Current status of newborn screening worldwide: 2015. Semin. Perinatol..

[157] Janeckova H., Hron K., Wojtowicz P., Hlidkova E., Baresova A., Friedecky D., Zidkova L., Hornik P., Behulova D., Prochazkova D. (2012). Targeted metabolomic analysis of plasma samples for the diagnosis of inherited metabolic disorders. J. Chromatogr. A.

[158] Dercksen M., Koekemoer G., Duran M., Wanders R.J.A., Mienie L.J., Reinecke C.J. (2013). Organic acid profile of isovaleric acidemia: A comprehensive metabolomics approach. Metabolomics.

[159] Ostermann K.M., Dieplinger R., Lutsch N.M., Strupat K., Metz T.F., Mechtler T.P., Kasper D.C. (2013). Matrix-assisted laser desorption/ionization for simultaneous quantitation of (acyl-)carnitines and organic acids in dried blood spots. Rapid Commun. Mass Spectrom..

[160] Fan M., Sidhu R., Fujiwara H., Tortelli B., Zhang J., Davidson C., Walkley S.U., Bagel J.H., Vite C., Yanjanin N.M. (2013). Identification of niemann-pick c1 disease biomarkers through sphingolipid profiling. J. Lipid Res..

[161] Reinecke C.J., Koekemoer G., Westhuizen F.H., Louw R., Lindeque J.Z., Mienie L.J., Smuts I. (2011). Metabolomics of urinary organic acids in respiratory chain deficiencies in children. Metabolomics.

[162] Smuts I., Westhuizen F.H., Louw R., Mienie L.J., Engelke U.F.H., Wevers R.A., Mason S., Koekemoer G., Reinecke C.J. (2012). Disclosure of a putative biosignature for respiratory chain disorders through a metabolomics approach. Metabolomics.

[163] Venter L., Lindeque Z., Jansen van Rensburg P., van der Westhuizen F., Smuts I., Louw R. (2014). Untargeted urine metabolomics reveals a biosignature for muscle respiratory chain deficiencies. Metabolomics.

[164] Wikoff W.R., Gangoiti J.A., Barshop B.A., Siuzdak G. (2007). Metabolomics identifies perturbations in human disorders of propionate metabolism. Clin. Chem..

[165] Auray-Blais C., Boutin M., Gagnon R., Dupont F.O., Lavoie P., Clarke J.T. (2012). Urinary globotriaosylsphingosine-related biomarkers for fabry disease targeted by metabolomics. Anal. Chem..

[166] Shlomi T., Cabili M.N., Ruppin E. (2009). Predicting metabolic biomarkers of human inborn errors of metabolism. Mol. Syst. Biol..

[167] Mutze U., Beblo S., Kortz L., Matthies C., Koletzko B., Bruegel M., Rohde C., Thiery J., Kiess W., Ceglarek U. (2012). Metabolomics of dietary fatty acid restriction in patients with phenylketonuria. PLoS ONE.

[168] Pan Z., Gu H., Talaty N., Chen H., Shanaiah N., Hainline B.E., Cooks R.G., Raftery D. (2007). Principal component analysis of urine metabolites detected by nmr and desi-ms in patients with inborn errors of metabolism. Anal. Bioanal. Chem..

[169] Gertsman I., Gangoiti J.A., Barshop B.A. (2014). Validation of a dual LC-HRMS platform for clinical metabolic diagnosis in serum, bridging quantitative analysis and untargeted metabolomics. Metabolomics.

[170] Miller M., Kennedy A., Eckhart A., Burrage L., Wulff J., Miller L.D., Milburn M., Ryals J., Beaudet A., Sun Q. (2015). Untargeted metabolomic analysis for the clinical screening of inborn errors of metabolism. J. Inherit. Metab. Dis..

[171] Aygen S., Dürr U., Hegele P., Kunig J., Spraul M., Schäfer H., Krings D., Cannet C., Fang F., Schütz B. (2014). NMR-based screening for inborn errors of metabolism: Initial results from a study on turkish neonates. JIMD Rep..

[172] Goodacre R., Broadhurst D., Smilde A.K., Kristal B.S., Baker J.D., Beger R., Bessant C., Connor S., Capuani G., Craig A. (2007). Proposed minimum reporting standards for data analysis in metabolomics. Metabolomics.

[173] Chitayat S., Rudan J.F. (2016). Chapter 10—Phenome centers and global harmonization. Metabolic Phenotyping in Personalized and Public Healthcare.

[174] Kohler I., Verhoeven A., Derks R.J., Giera M. (2016). Analytical pitfalls and challenges in clinical metabolomics. Bioanalysis.

[175] Alyass A., Turcotte M., Meyre D. (2015). From big data analysis to personalized medicine for all: Challenges and opportunities. BMC Med. Genom..

[176] Tarailo-Graovac M., Shyr C., Ross C.J., Horvath G.A., Salvarinova R., Ye X.C., Zhang L.H., Bhavsar A.P., Lee J.J., Drogemoller B.I. (2016). Exome sequencing and the management of neurometabolic disorders. N. Engl. J. Med..

[177] Sahoo S., Franzson L., Jonsson J.J., Thiele I. (2012). A compendium of inborn errors of metabolism mapped onto the human metabolic network. Mol. Biosyst..

